# Disability-inclusive community development: A case of a community garden in Limpopo province in South Africa

**DOI:** 10.4102/ajod.v11i0.850

**Published:** 2022-01-06

**Authors:** Brian Tigere, Theresa Moyo

**Affiliations:** 1 Turfloop Graduate School of Leadership, Faculty of Management and Law, University of Limpopo, Polokwane, South Africa

**Keywords:** disability-inclusive community development, persons living with disabilities, community development, community gardens, disability and development, livelihoods, accessibility

## Abstract

**Background:**

Persons with disabilities living in rural areas are marginalised and excluded in most developmental initiatives in South Africa. They face many economic, political and social problems; hence, improving their quality of life is a daunting and challenging task which needs interventions from both the state and non-governmental stakeholders.

**Objectives:**

This study aimed to examine the role played by community gardens in rural Limpopo province in uplifting the lives of persons living with disabilities as well as their communities as a whole. Its main objectives were to assess the social and economic benefits they have provided to this group of people.

**Method:**

A qualitative research design was used for this study. Twenty-one participants were identified through purposive sampling. They were made up of people with disabilities, officials from Departments of Agriculture and Social Development. Face-to-face interviews were used to collect data which was analysed thematically.

**Results:**

Key results were that community gardens have contributed to the economic and social well-being of persons with disabilities. They have assisted them with income to supplement their social grants. They also created jobs for their members and contributed to improved livelihoods of their families.

**Conclusion:**

The study demonstrated that people with disabilities are capable people who, if given the necessary support, can transform their livelihoods both socially and economically. The study recommends that a disability access audit be conducted to resolve the accessibility challenges of the garden.

## Introduction

Community development programmes are widely seen as a common strategy in uplifting the standards of living in poor rural and urban communities in the world and Africa in particular. Swanepoel and De Beer (2012:23) describe community development as a social transformation that involves changes in the awareness, motivation and behaviour of individuals and in the relations between individuals as well as between groups within the society. They emphasise that these changes have to originate from or be rooted from within individuals or groups rather than being externally driven. This insider conceptualisation is shared by Sharma (2013:183) who argue that community development has to be at grassroots level because that is where communities reside. He also qualifies ‘change’ from a broad perspective, that, essentially, consists of economic, social and cultural improvement of a community. Green and Haines (2015:3) articulate the objectives of community development in terms of eradicating unemployment and poverty and also reducing the inequalities in power relations amongst different classes of people in the society in order to reduce inequalities. The definition by Ndlovu (2012:8) emphasises community development as a joint effort between communities, government and other stakeholders, with the objective of improving the socio-economic and cultural conditions of a community. Ndlovu (2012:14) also adds that the process has to be participatory in terms of ensuring that the people who are expected to benefit from the process, should be fully involved at all levels, such as for example, the origination, design and implementation of a community initiative.

Each of the above definitions have merit in that they bring to the table, critical components of community development. Integrating the different elements, community development is a participatory, multi-stakeholder process which is defined and driven by the community for the purpose of achieving transformative socio-economic and cultural change for its members. This integrated view enabled the authors to critically analyse the impact of community gardens on people living with disabilities.

Certain groups such as persons living with disabilities are often marginalised from such initiatives. Therefore, they will not get their intended benefits unless deliberate efforts are made to ensure their full participation and integration of their interests into the design and implementation of the community development initiatives.

## Aim and objectives

The aim of the study was to examine the role played by community gardens in rural Limpopo province in uplifting the lives of persons living with disabilities as well as their communities as a whole. Its main objectives were to assess the social and economic benefits they have provided to this group of people. Another objective was to find out the role of these gardens in alleviating poverty, on a sustainable basis, amongst persons with disabilities.

There were two key questions that guided the analysis. The first was as follows: Is the community garden project model an effective strategy for community development with a focus on people living with disabilities? The second question was the following: Is the community garden model a sustainable approach for the development of persons living with disabilities?

The area in which the study was undertaken has a small population of 108 321 (Statistics South Africa [Stats SA] 2016). However, the number of persons with disabilities living in the area is not known.

## Community development projects

According to Westoby (2014), community development projects (CDPs) aim to improve the social, environmental, and economic situation of people especially in rural communities. In South Africa, these projects took different forms. Originating in the colonial era, they included agriculture, animal husbandry, public health, education, as well as small scale industries (Singh & Chudasama 2020). As observed by De Beer and Swanepoel (2012:3), their colonial legacy is evident in that essentially, they were designed more as measures for poverty alleviation rather than for achieving socio-economic transformation. Generally, income generating projects focus on poor communities, with the aim of creating opportunities to the local population. Chambers and Conway (1992) in United Nations Development Programme (UNDP 2017:3) maintain that community development initiatives are a source of sustaining improved livelihoods amongst community members. Olivier (2019:21) also opined that ownership and partaking in a community garden makes important contributions to improved and sustainable livelihoods including enhanced social status in a community attributed through financial income.

### Disability as an issue in development

Disability has been at the periphery of developmental policy formulation and implementation (Dube & Charowa 2005:7). This has led to both formal and informal discrimination and exclusion of persons with disabilities in developmental activities (Grech 2015:31). Formal discrimination occurs when developmental policies are silent on disabilities or do not include persons with disabilities in their formulation. Informal discrimination happens when society discriminates against persons with disabilities by preventing them from participating in community development. This may include inaccessible projects which directly hampers the participation of persons with disabilities.

The United Nations Convention on the Rights of Persons with Disabilities (UNCRDP) of 2006, of which South Africa is a signatory, acknowledges that persons with disabilities have the same rights like any other person. The Convention on the Rights of Persons with Disabilities (CRPD) bases its notion on articles which gives liberties in rights to education, participation, health as well as access to equal opportunities (Grech 2015). Human rights advocates claim that persons with disabilities are entitled to the same liberties as those without disabilities, without exceptions (Johnstone et al. 2012).

As observed by Rohwerder (2018), many African countries have people living with disabilities, often caused by communicable diseases, poverty, famine and armed conflicts. The situation of people living with disabilities is compounded by social prejudices and some cultural beliefs and attitudes that denigrate them. Consequently, they are often left out of development (Adeola 2015:227). However, there are no accurate and recent statistics on the prevalence of disability in Africa. South Africa is one of the few countries with detailed statistics on this. Out of its 57 million citizens, about 5.7% are said to be living with a disability in South Africa (Stats SA 2016).

The inclusion of persons with disabilities is a topical issue in Africa’s development because generally, they tend to experience discrimination and marginalisation from both community and international development efforts (Palmer 2012:217). Their development is being inhibited by the kind of approaches which countries adopt in relation to them. In many African countries, the approach tends to be welfarist rather than developmental. They implicitly portray disability as incapacity that can only be addressed through social welfare. A popular perception is that people with disabilities just require handouts and support because they are not able to contribute meaningfully to development (Tigere & Moyo 2019:6). Accordingly, they are not empowered to fully develop themselves and move out of poverty. In South Africa, for instance, the state provides a disability grant because most of the people with disabilities are unable to secure formal jobs. Unfortunately, some grant recipients have become dependent on the grant, instead of pursuing more independent sources of livelihood. This welfarism is one of the obstacles to the full empowerment of persons with disabilities. This observation is supported by Lopes (2019) who argues that disability grants in South Africa have created a perception that persons with disabilities are better off with the financial handouts from the state. The social model which the study utilised regards disability to be a result of the way society is organised. Beaudry (2016:215) advances a social model of disability which is a framework that conceptualises disability as a socially constructed problem. The author argues that persons with disabilities are as human as any able-bodied person and like everyone else, they should be fully integrated into society. Anastasiou and Kauffman (2013:442) advocate the need to address the economic, environmental and cultural barriers encountered by people who are viewed by others as ‘having some form of impairment whether physical, sensory or intellectual’.

### Disability friendly community development projects

Westoby (2014) is of the view that CDPs are aimed at improving communities by planning and executing developmental programmes. They focus on improving the social, environmental, and economic situation of people especially in rural communities. In South Africa, CDPs came in different ways which included agriculture, animal husbandry, public health, education, as well as small scale industries which had a colonial legacy (De Beer & Swanepoel 2012:3). This view is corroborated by Ndlovu (2012:13), who states that CDPs in South Africa took the shape of income generating activities such as home economics, sewing, wood work and particularly gardening. It is quite clear from historical evidence that the CDPs were designed to alleviate poverty but not really change the status quo in terms of racial inequality which was rooted in political, economic and social inequality.

The origin of disability friendly CDPs in South Africa also dates back to the apartheid era. The disability CDPs were viewed as accessible places of income generating activities such as gardening, woodwork or sewing and accommodate every disability of a person. The attempt was to take into account the peculiar needs of persons with disabilities. The model was based on the notion that persons with disabilities have faced historic barriers in CDPs in which most able-bodied persons participate (Johnstone et al. 2012:109). During the apartheid era, separate development for races was established. The black population which accounted for 80% citizens, suffered from the established oppression resulting in extreme poverty. This led to a crisis where marginalised groups such as persons with disabilities suffered greatly (Fish Hodgson 2018). In essence, they were also caught up in a deeply divided and unequal society (Schnitzler 2020). Like most black people, black people with disabilities were marginalised and excluded from the mainstream economy as compared to their privileged white counterparts. The experiences were therefore different between black and white disabled persons.

With the advent of democracy in 1994, CDPs became more organised and funded through firstly the department of Social Welfare and Local Government and then later the Department of Social Development as a poverty alleviation tool (Shah 2016). Unfortunately, although this model has been used as a strategy of community development without transforming it so that it addresses the fundamental root causes of inequality in South African society, causes which include but are not limited to inequality in access to productive resources, education and skills as well as access to finance.

### A case of a community garden

The main objective of this study was to examine whether community gardens for persons with disabilities, as a strategy of community development, have made any significant impact in enhancing inclusive community development. The persons living with disabilities in the study area decided to start a community garden mainly because of the benefits they saw. These benefits include a source of income, food security and also improving their livelihoods. They also saw it fit to do gardening because their functional limitations would suit agricultural activities. The project was started together with community members. It is registered under the *Non-Profit Act* of 1997. The able-bodied community members were drafted to work in administration as they were perceived to be knowledgeable of the management of projects. Governmental departments play a supporting role for the community gardening. For instance, the Department of Social Development is the custodian of the programme on Persons with Disabilities and it provides minimal financial subsidy to cater for the administrative costs such as office equipment, food and electricity. The land was given by the local traditional authority office and is owned by the persons with disabilities who own the community garden. The group has title deeds for the land as reflected on the nonprofit organisation (NPO) certificate. Under the *NPO Act* of 1997, any organisation which registers under the act must be run by a board committee which appoints the management to run the day to day business. In the case of the community garden, the board is non-functional and is actually controlled by the management. This is a common problem in many projects of similar nature.

This study was motivated by an interest to assess the impact on project beneficiaries, of a community garden that was initiated by people living with disabilities. This was a registered NPO project in which the land was allocated by a traditional authority. In our opinion, it had the potential to empower the members because, unlike traditional CDPs described above, the ownership of a resource like land and control by the members, were fundamental factors in achieving economic and social transformation of the members.

## Research methods and design

A qualitative research approach was utilised for the study because the researchers were interested in understanding the impact of these projects from the perspective of the persons living with disabilities. According to Lewis (2015:473), the qualitative research approach allows researchers to gain an in-depth understanding of human behaviour and factors that govern it. The experience survey was used in the study. Kothari (2004:36) explain this type of survey as a survey of people who have had practical experience with the problem to be studied.

The population of the study was 66 community members who are engaged in community gardening. The study was only interested in the community garden which caters for persons with physical disabilities. Thus, the study sample was selected from persons with physical disabilities working at a community garden in some local municipality in Limpopo province of South Africa. It was also important to include two management personnel because they had useful insights on the day-to-day running of the garden. The sample size was 21 participants (see Table 1). Nineteen were persons with disabilities. The inclusion criterion was that participants should be persons with disabilities (paraplegics, hemiplegics) of all genders. For those from outside the gardens, there were two persons from the community, namely the Project Manager and the Administrator. The officials were selected on the basis that they were working closely with the community garden projects.

**TABLE 1:** Biological profile of the study participants.

Pseudonym	Age	Sex	Impairment	Educational qualifications	Training in gardening
Participant 1	26	Male	Paraplegia	Matric	n/a
Participant 2	31	Female	Hemiplegia	Grade 10	n/a
Participant 3	33	Male	Paraplegia	Matric	n/a
Participant 4	25	Female	Paraplegia	Grade 11	n/a
Participant 5	28	Female	Paraplegia	Grade 10	n/a
Participant 6	25	Female	Hemiplegia	Grade 11	n/a
Participant 7	44	Female	Paraplegia	Grade 11	n/a
Participant 8	31	Female	Paraplegia	Matric	n/a
Participant 9	33	Female	Hemiplegia	Matric	n/a
Participant 10	36	Male	Hemiplegia	Matric	n/a
Participant 11	31	Male	Hemiplegia	Grade 11	n/a
Participant 12	28	Male	Paraplegia	Grade 10	n/a
Participant 13	29	Female	Paraplegia	Matric	n/a
Participant 14	31	Male	Hemiplegia	Matric	n/a
Participant 15	59	Male	Paraplegia	Grade 8	n/a
Participant 16	61	Female	Paraplegia	Grade 8	n/a
Participant 17	43	Male	Hemiplegia	Grade 10	n/a
Participant 18	47	Male	Hemiplegia	Grade 10	n/a
Participant 19	53	Female	Paraplegia	Grade 8	n/a
Participant 20	26	Female	n/a	Matric	Yes
Participant 21	31	Male	n/a	Matric	Yes

*Source*: Field survey, 2020

The researchers utilised purposive sampling. Therefore, the researcher decided on what need to be known and set out to find people who can and are willing to provide the information by virtue of knowledge and experience. After seeking the permission to conduct the study in the area and also after explaining the details of the study to the participants in the community garden, the researcher purposefully selected the 21 in such a way as to include males and females, the young and the old. Face-to-face interviews were utilised to gather data. Kothari (2004) opined that face-to-face interview is a data collection method whereby a researcher communicates directly with a participant to solicit information guided by a questionnaire. The choice of this method was that it allowed for confidentiality and privacy. Consistent with the principles of ensuring no harm to research participants, it was important to protect each participant from any possible harm that might arise as a result of expressing their true opinions on the issues that were to be discussed.

Because this was a qualitative case study design, thematic analysis was applied to analyse the data collected from the face-to-face interviews. Interview responses were recorded by video after seeking permission from the participants. Firstly, data was translated from the original recordings. Translation (from Se Sotho to English) was done because most interviews were carried out in the native language. Kothari (2004:122) defines this stage as editing whereby a careful scrutiny of the data recorded in interviews is done to assure that the data is accurate. They were then transcribed into a word document. Secondly, data was coded to identify key factors such as words, sentences and meanings. Lewis (2015) is of the view that coding is the link between data collection and explanation of its meaning. MAXQDA software was used for the coding process. The transcribed interview data was imported into MAXQDA as a word document. The researchers went through the text paragraph by paragraph and attached labels or codes to the text, based on their understanding of what the participants were saying. These codes were divided into main (parent) and sub-codes, the latter reflecting that they were related to the main idea in a text. These codes were then grouped together to identify emerging themes from the data. These themes became the basis for the presentation of results and discussion. The software also made it easy for the researchers to extract direct quotes from some participants on the issues that we thought were central to the focus of the study.

Our understanding of thematic analysis was informed by the work of Williams and Moser (2019) who define coding in qualitative research as being comprised of ‘processes that enable collected data to be assembled, categorized, and thematically sorted, providing an organized platform for the construction of meaning’. They also explain that coding reveal themes that are embedded data and that this allowed for meaning to be negotiated, codified, and presented.

## Trustworthiness

Denzin and Lincoln (2011:44) are of the view that, ‘in any study, the findings must be believable, consistent, applicable and credible if they are to be useful to readers and other researchers’. To ensure trustworthiness of the study findings, credibility, applicability, consistency and neutrality criteria were utilised. Shenton (2004:35) notes that ‘credibility refers to demonstrating that the inquiry in a study was conducted in such a manner that the subject was accurately identified and described’. To ensure credibility, the qualitative methodology utilised in the study was clearly laid down. In the study, face-to-face interviews were utilised after the participants were chosen through purposive sampling. Transferability was achieved in the study through giving a detailed background of the study area which is Molemole Local Municipality of Limpopo province. This allows readers and other researchers to relate the findings with other similar study areas. Shenton (2004:36) opined that, ‘dependability entails that researchers should at least strive to enable future investigators to repeat the study’. Dependability was achieved through giving a detailed research design which gave tools utilised for data collection as well as its interpretation. Conformability refers to the objectivity of the study during data collection and analysis (Denzin & Lincoln 2011:45). Conformability in the study was achieved through safekeeping of the data collected for other researchers to agree or corroborate with the findings when required.

### Ethical considerations

Several ethical obligations were met by the researchers in this study. Permission was sought from the university which the study had jurisdiction. An Ethics Clearance Certificate was issued as proof of approval for the study to be carried out. The study was approved by Turfloop Research Ethics Committee of University of Limpopo, reference number: TREC/204/2018/PG. Confidentiality was also preserved in the study through the use of pseudonyms to identify the participants. Informed consent as well as voluntary participation was ensured through the signing of consent forms by all the participants. As a way of ensuring voluntary participation, the participants were told that they can withdraw at any stage of the study without any consequences.

## Results

The results emanate from rich qualitative data gathered from interviews conducted with 21 participants. The key questions posed at the beginning of the study guided the presentation and discussion of results. The presentation is divided into three sections: (1) Is the community garden project model an effective strategy for community development with a focus on people living with disabilities?; (2) Is the community garden model a sustainable approach for the development of persons living with disabilities? and, (3) Discussion and implications of findings.

### Is the community garden project model an effective strategy for community development with a focus on people living with disabilities?

In this part of the presentation of findings, the researchers report on the nature of the community gardening projects, how they are managed, how they are operating and what benefits (if any) are being realised by persons living with disabilities. The selection of the sub-headings was informed by the identified themes after coding interview transcripts. Some key themes were gardening as a source of income; a source of livelihoods; a source of employment. Other themes were as follows: management, decision-making, participation, social cohesion, technical design of gardens, sustainability. These guided the presentation of findings.

#### Nature of gardening projects at the case study community garden

The findings of the study revealed the importance of integrating the interests of persons living with disabilities in CDPs. Frustrated by their exclusion from some of the development initiatives in their areas, the community of persons living with disabilities decided to establish a community garden with the design features that were suited for them.

The community garden under the study has many gardening projects which are being undertaken simultaneously. They involve what the participants highlighted as the green projects, where all plant production takes place. The green project is subdivided into the vegetable section, which grows a variety of fresh vegetables all year round such as cabbages, carrots as well as two varieties of spinach, namely savoy and Chinese. These two varieties of spinach are grown throughout the year and are responsible for almost half of the income of the garden. Beetroot and butternuts are also grown at the garden and are mostly seasonal, with extensive growth in summer for the December holiday markets. The last project of the garden is the animal husbandry section where chicken rearing takes place. Layers for eggs and broilers for meat are kept all year round and bring a steady income to the centre. The different types of gardening projects are shown in Table 2.

**TABLE 2:** Different types of gardening projects as well as produce at the case study community garden between 2018 and 2019.

Project	2018	2019
**Green project**		
Cabbages	155 heads	201 heads
Spinach (Savoy and Chinese)	204 bunches	236 bunches
Beetroot	105 bunches	110 bunches
Butternut	76 pockets	70 pockets
Maize	25 bags	31 bags
Carrots	96 bunches	108 bunches
**Animal husbandry**		
Chicken	260 birds	300 birds
Egg production	65 crates	78 crates

*Source*: Field survey, 2020

#### Origin of the idea of community gardens

The interviews with the participants confirmed that the idea of community gardens was started by the persons with disabilities who reside in the study area when they saw a need for income generation amongst themselves. This is reflected in the following responses of some participants:

‘We just came up with the idea of a gardening project after realising that we are just sitting home and doing nothing.’ (Participant 5, Female, 28 years)

‘It was at a forum for us people with disabilities that we thought of supplementing our disability grants with an income generating initiative. Gardening was approved by the majority of us.’ (Participant 15, Male, 59 years)

#### Projects as a source of income

The participants explained that on average, they get roughly R1000.00 per month each. However, there are factors which contribute to changes in income such as planting and harvesting times. For instance, in December, income is significantly higher because of sales of butternuts, carrots as well as beetroots purchased by social ‘stokvel’ groups as well as organisations for their year-end functions. Therefore, entrepreneurship and rural development is promoted. The participants pointed out that their economic livelihoods have improved to a greater extent since their participation in the community garden.

One of the research participants stated the following:

‘The produce is sold at the local market (schools and government departments) and some to the Polokwane vegetable market. The income supplements my disability grant to some extent.’ (Participant 15, Male, 59 years)

Another participant explained:

‘We receive some of the payments or stipends after the sale of the produce. Although it is not enough, it helps a bit to improve my financial conditions since sitting at home doing nothing is not good at all. Half a loaf is better than nothing.’ (Participant 3, Male, 33 years)

To some extent, the participants were able to reduce their dependence on other people because they do not have to purchase vegetables such as cabbages, spinach and tomatoes from supermarkets and shops. This acts as a saving because the finances are used for other things; hence, expenses on food are reduced. Galhena, Freed and Maredia (2013:9) agree that generally, economic benefits of community gardens go beyond food and nutritional security at household level, especially to disadvantaged groups within communities such as the elderly, women and persons living with disabilities. The researchers noted that incomes were also constrained by limited access to markets. Access to transport to enable project members to sell fresh produce at bigger places such as busy malls and taxi ranks is likely to increase their income.

From our understanding of development as explained in the literature review, many members of this gardening project are able to get an income, the amounts are so low, they serve only as supplements to their disability grant. An income of R1000.00 is way below the poverty line in South Africa. So, the members of this project would have to continue to depend on the disability grant. This demonstrates that the community garden project as it currently operates in the context of the case study is not a sustainable strategy for community development.

#### Livelihoods

To a certain degree, there was livelihood advancement in terms of incomes received by the participants since they joined the garden. Some of them explained that:

‘I am now able to get material things to improve my livelihood and that of my family since I am getting financial rewards after the sale of the garden produce. It is better and supplementing my disability grant.’ (Participant 6, Female, 25 years)

Another explained that:

‘Formal employment was really difficult and elusive to come by due to my disability. Ever since joining this community garden since the last 2 years, there has been a steady flow of income which has to some extent improved my situation and that of my family.’ (Participant 7, Female, 44 years)

It is quite clear from the responses that whilst the participants are receiving income from the projects, the money received is actually not enough for their needs. They seem to accept that nonetheless, having some income is better than not having anything at all. The fact that the project is not generating enough income for the participants raises questions about the capacity of the project as a long-term solution to their development needs.

#### Indigenous knowledge preservation of gardening and persons living with disabilities

Persons with disabilities have developed a culture of doing gardening in ways which are unique to them as a group of special abilities. Their functional abilities on gardening tasks have made them master and build their own indigenous knowledge on agricultural activities. Because they are not able-bodied, their disabilities have made them to adjust and be innovative in the way they do gardening. This was reflected in the following responses of some participants:

‘We are doing the activities such as weeding according to our abilities, that is, we have to be innovative. For instance, most of the time we weed, we do not use a wheelchair but rather crawl along the bed which is faster and efficient to us. We share these innovation skills to our persons living with disabilities peers and transfer the knowledge to future generations.’ (Participant 11, Male, 31 years)

‘When we fertilise, we do the broadcast method which is easier and efficient for us as people with disabilities.’ (Participant 13, Female, 29 years)

Other participants also explained that one of the suggestions they would have liked to make if the management was receptive, was that the vegetable beds should be constructed such that they could be at the same level as a person in a wheelchair. That way, it would be easier for them to cultivate the crops. Furthermore, wider spacing of beds would improve wheelchair access and thus, enhance the efficacy of their work.

This discussion demonstrated the creative capacity of persons with disabilities. The innovative techniques they do have at their disposal to adapt their own abilities to tasks at hand no doubt add to indigenous knowledge.

Scholars such as Ndlovu (2012:56) have also argued that the preservation of indigenous knowledge is important for community gardens.

#### Employment

Agriculture is predominantly labour-intensive and therefore requires manual labour. Employment creation is a major positive factor to the persons living with disabilities at the community garden. Although it is not formalised through employment contracts, job opportunities have been created informally. The persons living with disabilities are employed informally to work in the garden. They receive a monthly stipend in accordance with the sales of the produce. Although the financial rewards are not much, the participation in the activities and its administration such as duty sheets act as employment. Some of the research participants had this to say:

‘Although I do not have a contract, my work hours are clocked everyday hence this leads to my stipend pay out at the end of the month.’ (Participant 6, Female, 25 years)

‘My duty sheet denotes my responsibilities such as weeding, feeding the chickens and if I do not do that I am breaking my employment code.’ (Participant 15, Male, 59 years)

‘By virtue of the fact that the community garden belongs to us persons with disabilities, this provides us with a sense of ownership and that we are doing something meaningful with our lives.’ (Participant 7, Female, 44 years)

The responses acknowledge that although it is not formal, the community garden acts as an employer to them. Ownership of the community garden provides a sense of identity and self-gratification to do something positive and meaningful with their life.

#### Strengths of the community garden

The community gardens project had some positive impacts. One is that they were able to induce livelihoods for persons living with disabilities. This is because of the fact that income derived from the sales of the produce provided them with a salary. The project also provided a financial backbone to them as it supplemented the monthly disability grant provided by the state. The project also fosters social cohesion amongst its members.

The project also enhanced social capital, an important component in the sustainable livelihoods approach. The concept supports this notion that groups of people are brought together to increase their social functioning. This was brought up by one participant who shared her experience in the following words:

‘At the community garden, it is not only about cultivating vegetables. It is a place where we meet as a community of persons living with disabilities and discuss issues which affect us in the larger community. This assists us to improve our social and emotional wellbeing.’ (Participant 4, Female, 25 years)

Thus, apart from economic benefits, the gardens have a positive psycho-social effect in terms of improving social and emotional well-being of the participants.

### Is the community garden model a sustainable approach for the development of persons living with disabilities?

A critical issue in community development is sustainability of any development intervention. Shah (2016) is of the view that sustainability in community projects has to focus on a number of factors such as ownership and control of the project by members, participation in decision-making, fairness and equity in the distribution of benefits amongst members, size of income generated, and impact on the environment. Abiona and Bello (2013) saw that participation of the indigenous is key to promote sustainability of community projects.

In this section, we present the results on some of these critical issues on sustainability. These focus on the ownership and control, management, decision-making and participation, technical design of the gardens and social cohesion amongst group members.

#### Ownership and control of the community garden

The participants made it clear that the community garden was a registered NPO and that the land was given by the local traditional authority. So, the persons living with disabilities actually own it. However, because most of them had low levels of literacy and no skills in the management of such projects, they outsourced from the more able-bodied persons from the community. The interviews indicated that there was a rift between the managers and those who were treated as employees in the projects. This rift is discussed under the following section on management and decision-making. This suggests that although they own the land, they actually do not have control over it. This situation is likely to undermine long-term sustainability of the project.

#### Management, decision-making and participation

The management of the community garden comprises of two individuals who are the project manager as well as the administrator. Both of these two personnel are not disabled and the researchers observed that it poses a serious issue in the inclusiveness of persons living with disabilities in designing, planning and coordination of activities. They are the ones who are only trained in community gardening.

The project manager explained that:

‘The community garden is funded by the department of Social Development under the directorate of Persons with Disabilities and old age. Since we are provided with a small financial subsidy, the social workers come sporadically for monitoring but operational issues are left to us since we are a nonprofit organization (NPO). We as the management are responsible for the day-to-day decisions.’ (Participant 21, Male, 31 years)

The management is solely responsible for making operational decisions. The problem, however, was the exclusion of persons living with disabilities, who are the custodians of the garden, from decision-making processes. To confirm this, some participants who are persons living with disabilities explained that:

‘We are not consulted when decisions about our work are done. Issues like tasks and payments are not discussed openly with us. That compromises some of us since the schedule sometimes does not take into account our abilities.’ (Participant 18, Male, 47 years)

‘We do not have a representative of persons living with disabilities in the management and this creates serious challenges since no one can stand for us.’ (Participant 8, Female, 31 years)

‘We are just left put on key decisions which affect our work and the gardening. The management does not involve us mostly in issues which affect us directly.’ (Participant 13, Female, 29 years)

The responses from the participants suggest that the management approach was top-down, leaving no room for full participation of members. According to Swanepoel and De Beer (2012:78), participation is a process and implies collective activity of interested or concerned people in achieving a jointly determined goal. Nel (2015:514) corroborates this view when he argues that real participation is where community members share fully and have an equal voice in all decision-making and efforts directed towards change. Clearly, participation was lacking in these projects. This is one of the factors that limit the potential of the projects as a sustainable solution to the development challenges faced by persons with disabilities.

#### Technical dimensions of the project: Are they fit for purpose?

The social model of disability suggests that the environment should be accessible to all persons with disabilities to participate in economic, social and political activities (Grech 2015). In this study, it was observed that the community garden is not 100% accessible to wheelchair users. Thus, a technical challenge that might impede long-term sustainability lies in the *poor technical design of the gardens.* Some participants explained that they were not included to a large extent in the designing of projects at the community garden. They complained that:

‘The management does not involve us in the most important things such as the design of beds in accordance to our disabilities.’ (Participant 11, Male, 31 years)

Another said that:

‘There is a lack of consultation and participation amongst us the disabled on critical issues of accessibility in accordance to our disabilities. The management consist of people who are not disabled hence they make a lot of mistakes.’ (Participant 19, Female, 53 years)

The lack of consultation of members resulted in some of the beds being too low for wheelchair users. This was pointed out by one participant:

‘The vegetable beds are sometimes too high or too low. They were constructed without proper consultations on our disabilities. For instance, as a paraplegic and using a wheelchair, I cannot work on other beds since they are too low.’ (Participant 16, Female, 61 years)

An official from one of the government departments responded to the researcher concerning the complaints from participants:

‘We try to have raised beds for our vegetables for easy access of persons living with disabilities when they are going on with their work. I do admit that we only construct these raised beds from what we think is right since we do not have any technical aspects of doing it.’ (Participant 21, Male, 31 years)

It was also observed that there were a few raised seedbeds which do not have the correct configuration for wheelchair users. Consistent with the principle of ‘reasonable accommodation’ which denotes to the accessibility of working environments for persons living with disabilities, a disability audit conducted by an accessibility specialist would have been necessary. According to Beaudry (2016:216), reasonable accommodation provides for the means and processes of making an environment workable by people living with disabilities. The issues of poor accessibility and low seedbeds could have been addressed if participation and consultation were done properly.

#### Social cohesion amongst group members

Egli, Oliver and Tautolo (2016) highlighted that social cohesion is important for the success of any project. Social cohesion is defined as strengths of relationships and the sense of solidarity amongst members of a group or community (Nettle 2014). In this case study, it was clear that there was no social cohesion. This was evident by the apparent rift between the managers and the participants who, as explained before, were persons living with disabilities.

One of the participants noted the following:

‘There are two distinctive camps within the garden. One of us was the real owner of the garden and the other is the management who are responsible for administration and we are not in harmony at all. The relationship is not that good.’ (Participant 9, Female, 33 years)

The persons living with disabilities were excluded from decision-making and did not have the opportunity to contribute their ideas on how the project could improve its performance. Whilst they own the land, they are not privy to important information such as sales, revenues generated by the project and expenditures on the project. The fact that they do not seem to have capacity to engage the managers also raises questions about the future of the projects. Thus, the lack of social cohesion threatens the sustainability of the project in the long-term.

## Discussion and implications of findings

The introduction of this article began by explaining the concept of development from the perspective of a number of authors. These included Swanepoel and De Beer (2012), Sharma (2013), Green and Haines (2015) and Ndlovu (2012), amongst others. Based on this and other literature, the authors interpreted community development as a participatory, multi-stakeholder process which is defined and driven by the community for the purpose of achieving transformative socio-economic and cultural change for its members. This understanding of community development provides a useful framework within which to evaluate the impact of the community garden project on persons living with disabilities.

Findings from the study indicate that there were some benefits enjoyed by the participants from the garden project. Some of the benefits were that the project was a source of employment for them. They also enabled them to earn a monthly salary which supplemented their disability grant. For Cumbers et al. (2018:135), gardening activities, though informal, usually provide working opportunities. Wise (2014:22) confirms that community gardening creates a sense of employment because income is derived, and split and shared on a timeous basis which is determined by the members. It can therefore be concluded that although employment at the community gardens is not as formal as signing employment contracts, it serves the purpose of work.

Some participants also indicated that the project gave them integrity and enhanced their self-worth. The projects have also increased the availability of food for persons living with disabilities and their households. The garden offers different vegetables and staples, which safeguard both food and nutritional security. Other studies also confirm this positive impact of such gardens. For example, Galhena et al. (2013:7) argues that community gardens are a source of income generation which empowers the less advantaged in the community through economic emancipation. Egli et al. (2016:351) also argue that community gardening is less cost intensive, requires fewer inputs, and is extremely important for people who have limited access to production inputs.

Other participants explained that the project allowed them to network and socialise with other people living with disabilities. This had a therapeutic effect on them. For some of the participants, the community garden stimulated innovative thinking as they tried new ways of doing gardening that suited their physical circumstances. Even though some of their suggestions for creating more disability-friendly gardens were never taken up by the management, they clearly showed that they are capable of innovative thinking that could contribute towards the development of indigenous knowledge.

Whereas Cumbers et al. (2018:134) view community gardens as strategies for creating sustainable and ethical forms of living whilst also offering alternative ways of community development. In the case that was studied, the project created an opportunity for an ethical form of living. However, there are questions on the sustainability of the projects. Whilst these findings clearly demonstrated some positive impacts of community gardens of persons living with disabilities, in terms of our conceptualisation of community development, the impact was limited. Firstly, it was evident that the incomes generated from the project were viewed more as a supplement to their disability grant rather than as an income which could actually wean them from the grant altogether. A critical component of development is sustainability. If a project does not generate enough income to sustain a member, then it is not a sustainable solution to poverty and underdevelopment. Secondly, even though the persons with disabilities own the land on which the project is operating and are actually registered as an NPO, it appears that there is a hidden dynamic where, because of their limited education and skills, they outsourced managerial functions to more able-bodied community members. There is some power dynamics that resulted in loss of control by the owners, the persons living with disabilities. The loss of control was so perverse that the owners were excluded from participation in decision-making particularly on critical matters such as information on sales, project expenditures and profitability.

The accessibility challenges raised by the participants also present a barrier towards sustainability of the projects in the long-term. The study found that most of the persons living with disabilities are either hemiplegic or paraplegic, and use assistive devices such as wheelchairs, crutches and special orthopaedic shoes. This therefore requires an accessible environment such that the persons living with disabilities are free to conduct their gardening activities. As explained by some participants, the physical environment within the project prohibited the movement of persons living with disabilities in either their workplaces or in societies in which they live. The challenge of access is echoed by Abdullahi and Ahmed (2017:54) who argue that persons with disabilities cannot easily move around within their environments because of the inaccessible nature of buildings, pavements, roads and transport systems. This is further corroborated by Rohwerder (2018) who confirms these findings when he explains that environmental obstructions limit the productiveness of persons with physical disabilities’ access to production processes. This, therefore, limits their full participation in economic activities.

The participants complained that even though they had some innovative ideas on how best to design disability-friendly garden beds, their voices were not heard because they were not represented in the management. All these facts show that the persons with disabilities were not actually empowered. Being reduced to serve as employees, they had no autonomy at all. Because some of the key features of meaningful community development are autonomy and empowerment, this project falls short of being developmental. According to Bryant and Chahine (2016:169), the purpose of community gardens is derailed because of the lack of participatory action amongst the members. This creates a sense of mistrust which can disrupt the whole project. Reynolds and Cohen (2016:91) share the same sentiments in that lack of equal voices in these projects diminishes decision making and efforts directed towards change. In essence, participation amongst members is key because collective action brings better ideas which will lead to successful and sustainable community gardens.

## Conclusion and recommendations

The study has demonstrated the important role which community gardens can play in improving the livelihoods and welfare of people living with disabilities. Having taken the initiative to start a community gardening project, these people were able to generate incomes and improve access to food and nutrition. The gardens created jobs and also had other positive effects such as enhancing their dignity and self-worth. However, a major challenge relates to the lack of sustainability largely because of the dependence of the group on those who are able-bodied. Because of their own limited education levels and management experience, they co-opted able-bodied persons to take responsibility of the administration and management of the projects. However, the dynamics of unequal power relations between able-bodied and persons living with disabilities, amongst other factors, led to the exclusion of the latter from vital decision-making on payment of wages and also from disclosure of information on profitability of the projects. Thus, although persons with disabilities own the land on which the project is operating, they are actually disempowered. Furthermore, they feel that the exclusion has limited their ability to share innovative ideas on how the gardens can become more disability-friendly with respect to access and technological innovations. We therefore recommend that government departments and other stakeholders who are working to facilitate the development and empowerment of persons living with disabilities should implement measures that integrate the concerns and interests of such people to ensure that they benefit from such projects in a more sustainable and equitable way.

To resolve the accessibility challenge, it is recommended that a disability access audit be conducted for the purpose of informing the design and specifications to improve access to the garden environment.

It is recommended that the management structure should be representative of the sector that it serves, that is persons with disabilities. This will promote the disability movement motto of ‘Nothing for us without us’. To foster this approach, there is a need to commit to a rights-based approach to disabilities upon which inclusive development is seen as a right for persons with disabilities. It can therefore be concluded that the best way to promote disability inclusive community development is engaging with persons with disabilities as partners, expert advisors, active participants and beneficiaries. We recommend that the Departments of Agriculture and the Department of Social Development as well as other stakeholders working with persons with disabilities should consider adopting more participatory approaches so that the intended beneficiaries of projects are fully engaged in the affairs of the project.
